# Novel pathogenic mutations and skin biopsy analysis in Knobloch syndrome

**Published:** 2009-04-23

**Authors:** Oscar Suzuki, Erika Kague, Kelly Bagatini, Hongmin Tu, Ritva Heljasvaara, Lorenza Carvalhaes, Elisandra Gava, Gisele de Oliveira, Paulo Godoi, Glaucius Oliva, Gregory Kitten, Taina Pihlajaniemi, Maria-Rita Passos-Bueno

**Affiliations:** 1 Centro de Estudos do Genoma Humano, Departamento de Genética e Biologia Evolutiva, Instituto de Biociências, Universidade de São Paulo, São Paulo, Brazil; 2 Collagen Research Unit, Biocenter and Department of Medical Biochemistry and Molecular Biology, University of Oulu, Oulu, Finland; 3 Instituto de Ciências Biológicas, Universidade Federal de Minas Gerais, Belo Horizonte, Brazil; 4 Departamento de Física e Informática, Instituto de Física de São Carlos, Universidade de São Paulo, São Carlos, Brazil; 5 Departamento de Físico-Química, Instituto de Química de São Carlos, Universidade de São Paulo, São Carlos, Brazil

## Abstract

**Purpose:**

To facilitate future diagnosis of Knobloch syndrome (KS) and better understand its etiology, we sought to identify not yet described *COL18A1* mutations in KS patients. In addition, we tested whether mutations in this gene lead to absence of the *COL18A1* gene product and attempted to better characterize the functional effect of a previously reported missense mutation.

**Methods:**

Direct sequencing of *COL18A1* exons was performed in KS patients from four unrelated pedigrees. We used immunofluorescent histochemistry in skin biopsies to evaluate the presence of type XVIII collagen in four KS patients carrying two already described mutations: c.3277C>T, a nonsense mutation, and c.3601G>A, a missense mutation. Furthermore, we determined the binding properties of the mutated endostatin domain p.A1381T (c.3601G>A) to extracellular matrix proteins using ELISA and surface plasmon resonance assays.

**Results:**

We identified four novel mutations in *COL18A1*, including a large deletion involving exon 41. Skin biopsies from KS patients revealed lack of type XVIII collagen in epithelial basement membranes and blood vessels. We also found a reduced affinity of p.A1381T endostatin to some extracellular matrix components.

**Conclusions:**

*COL18A1* mutations involved in Knobloch syndrome have a distribution bias toward the coding exons of the C-terminal end. Large deletions must also be considered when point mutations are not identified in patients with characteristic KS phenotype. We report, for the first time, lack of type XVIII collagen in KS patients by immunofluorescent histochemistry in skin biopsy samples. As a final point, we suggest the employment of this technique as a preliminary and complementary test for diagnosis of KS in cases when mutation screening either does not detect mutations or reveals mutations of uncertain effect, such as the p.A1381T change.

## Introduction

Knobloch syndrome (KS; OMIM 267750) is an autosomal recessive disorder characterized by high myopia, macular abnormalities, vitreoretinal degeneration, retinal detachment, and occipital encephalocele [1-4]. The spectrum of clinical variability is not completely known due to the small number of cases reported in the literature [5]. Most KS cases are caused by null mutations in the *COL18A1* gene (chr21q22.3), which comprises 43 exons and transcribes three different isoforms by the use of two promoters and alternative splicing in the third exon [4,6]. The three encoded collagen XVIII proteins differ only by their signal peptides and by part of the N-terminal region of the NC11 domain. The short isoform (NC11–303) is transcribed from the first promoter, upstream of exon 1, and is encoded by exons 1, 2, 4–43. The intermediate (NC11–493) and long (NC11–728) forms are transcribed from the second promoter, upstream of exon 3, and they differ by use of an internal splice site within exon 3. Collagen XVIII is a component of basement membranes [7]; however, the abundance of its isoforms varies: the short isoform is predominant in most tissues, including heart, kidney, retina, and fetal brain, while the intermediate and long isoforms are highly expressed in the liver [3,4,6]. The C-terminal domain of type XVIII collagen can be cleaved to form endostatin, which functions as a potent angiogenesis inhibitor that influences endothelial cell proliferation, migration, apoptosis and tubulogenesis [8-11]. Endostatin binds to several extracellular matrix (ECM) components, including laminin-1, fibulin-1, fibulin-2, nidogen-2, perlecan, heparan sulfate, and fibronectin [12-14].

To date, 12 rare mutations have been described in KS patients with the typical clinical features of the disease [3-5,15-17]. All mutations, with the exception of a missense change (p.A1381T; numbered according to GenBank cDNA AF018081.1) of still unclear functional effect on the protein [18,19], are predicted to create premature stop codons. These mutations possibly lead to a lack of the protein, even though the complete lack of collagen XVIII has still not been demonstrated. *COL18A1* mutations are distributed along the gene in regions common to all known isoforms, except for c.12–2A>T, which only affects the short isoform. The description of pathogenic mutations in a larger number of KS patients and further functional analysis of the mutation p.A1381T may better characterize the spectrum of mutations along the *COL18A1* gene.

Screening the entire gene for mutations is still time consuming. We have previously shown that endostatin plasma measurements do not allow a precise diagnosis, as patients with null mutations had positive levels of this fragment [4], and no other methods to confirm KS diagnosis were tested. New strategies that might allow the screening of a larger number of patients, including those without the full phenotype of the syndrome, could help delineate the clinical variability.

In the present work we describe four new families with KS patients and the respective responsible mutations in *COL18A1*. Consistent with previous results, we find heterogeneity for mutation sites leading to this syndrome. We also assessed the immunohistochemical expression of type XVIII collagen in skin biopsies of KS patients carrying nonsense mutations to test the potential of this method as a screening tool for a larger number of patients. Finally, we evaluated in situ and in vitro effects of endostatin p.A1381T by testing skin biopsies for the presence of type XVIII collagen in KS patients carrying this mutation and evaluating the binding properties of the mutated endostatin to other ECM components.

## Methods

### *COL18A1* mutations analysis

Five KS patients were referred to the Centro de Estudos do Genoma Humano at the University of São Paulo. Two were siblings and the other three were not related. They presented with typical KS phenotypic characteristics: high myopia detected early in childhood (usually before 1 year of age) and occipital encephalocele, as described elsewhere [2,20]. No other alterations were observed.

We screened for mutations in the *COL18A1* gene by direct sequencing of exons and flanking intronic regions, as previously described [4]. All mutations were named according to the nomenclature suggested by den Dunnen and Antonarakis [21]. The numbering followed the short isoform (GenBank AF018082.1), except where indicated.

### Skin biopsies

After informed consent was obtained, 5 mm skin punch biopsies were taken from the forearms of four other KS patients, who were from two unrelated families. Clinical and molecular analysis of these patients has been described in detail by Suzuki et al. and Kliemann et al. [4,18]. Control skin samples were obtained from two patients that were undergoing surgery and had no suspicion of KS. Informed consent was also obtained from these patients. This project was approved by the Ethical Committee of the Institute of Biosciences, University of São Paulo (São Paulo, Brazil).

Tissue samples were fixed according to a procedure described elsewhere [22]. Briefly, samples were immediately fixed and cryosubstituted in a −70 °C solution of 80% methanol and 20% dimethyl sulfoxide for 5–7 days, transferred to −20 °C for 1–2 days, and then brought to room temperature. Samples were rinsed 3X in absolute ethanol and embedded in Paraplast Plus following standard protocols. In addition to maintaining morphological structure, this method also appears to preserve the antigenicity to a higher degree than aldehyde fixatives.

### Immunofluorescent histochemistry

Xylene was used to dewax 7 μm-thick sections. These were then rehydrated through a graded series of ethanol into phosphate-buffered saline (PBS; 137 mM NaCl, 2.7 mM KCl, 10 mM Na_2_HPO_4_, 2 mM KH_2_PO_4_, pH 7.4). Next, 1 mg/ml testicular hyaluronidase (Sigma, St. Louis, MO) in 50 mM acetate buffer, pH 5, was added at room temperature for 30 min. This was followed by several washes in PBS. Blocking was achieved using 2% BSA (BSA) in PBS at room temperature for 1 h followed by an overnight incubation at 4 °C with primary antibodies diluted in PBS containing 0.1% BSA and 0.01% Tween-20. A rabbit polyclonal antibody against type XVIII collagen (QH48.18, “anti-all”) which recognizes all three isoforms of type XVIII collagen [23] was used at a dilution of 1:300. Rabbit anti-human type IV collagen polyclonal antibody (Rockland, Gilbertsville, PA), an antibody against type IV collagen, was diluted 1:400 before it was used as a basement membrane marker. After several rinses in PBS, sections were incubated for 1 h at room temperature with 1:300 Cy3-conjugated goat anti-rabbit IgG secondary antibody (Jackson Laboratory, Bar Harbor, ME). After several rinses in PBS, sections were mounted in a mixture of 10% 1.0 M Tris-HCl, pH 9.0 and 90% glycerol and analyzed using a laser scanning confocal microscope (Zeiss 510META; Carl Zeiss AG, Oberkochen, Germany).

The distribution patterns and levels of expression of types IV and XVIII collagens were analyzed using pseudocolor images made with the thermo lookup table in the Confocal Assistant 4.02 freeware program. This tool utilizes gray-scale pixel values to generate pseudocolor images that allow the discrimination of small differences in the level of immunofluorescence.

### Molecular modeling

Human endostatin structure (1BNL) was obtained from the Protein Data Bank. the p.A1381T change was created and analyzed using GRASP [24] and Insight-II/Discover software (Molecular Simulations Inc., San Diego, CA).

### Recombinant endostatin production

The coding region of the endostatin domain was cloned into the pET-15b vector (Novagen, Madison, WI). p.A1381T endostatin was produced by PCR-based site directed mutagenesis.

Endostatin production and purification were based on a procedure published elsewhere [25]. Briefly, recombinant human endostatin expression in *E. coli* was induced using 1 mM IPTG (isopropyl-beta-D-thiogalactopyranoside) for 3 h. Cells were isolated by centrifugation and lysed by freeze thaw cycle in GUMCAC-0-buffer, which contained 6 M guanidine-HCl, 0.5 M NaCl, and 20 mM Tris-HCl, pH 7.9. This was followed by sonication in the presence of 15 mM β-mercaptoethanol. Lysate was loaded onto a ProBond™ column (Invitrogen, Carlsbad, CA) that had been previously equilibrated with URMCAC-0 buffer, which contained 8 M urea, 0.5 NaCl, and 20 mM Tris-HCl, pH 7.9. Endostatin was eluted with a gradient concentration of imidazole (0 to 500 mM) and dialyzed against 4 M urea, 0.1 M NaCl, 1 mM reduced glutathione, 0.1 mM oxidized glutathione, 20 mM Tris-HCl pH 7.9 (16 h at 4 °C). This was followed by a dialysis against 1 M urea, 0.1 M NaCl, 0.1 mM reduced glutathione, 0.01 mM oxidized glutathione, 20 mM Tris-HCl pH 7.9 (6 h at 4 °C), and a final dialysis step against PBS pH 6.9 (16 h at 4 °C). Human endostatin was then loaded onto a HiTrap-SP column (Amersham Biosciences, Uppsala, Sweden) and eluted using a NaCl gradient (0 to 1.5 M) in PBS pH 6.9. Buffer was exchanged by dialysis against 0.1 M NaCl and 20 mM Tris-HCl (pH 7.4) for 16 h at 4 °C.

### Binding assays

Surface plasmon resonance (SPR) assays were performed with a BIAcore® 3000 instrument (Biacore AB, Uppsala, Sweden). Extracellular matrix proteins, the laminin-1-nidogen-1 complex, perlecan, and fibulin were immobilized on CM5 sensor chips (Biacore AB) as described previously [26]. Mouse laminin-1-nidogen-1 complex and mouse perlecan were prepared from the mouse Engelbreth-Holm-Swarm tumor [27]. The recombinant microfibrillar component fibulin-1 was prepared following the procedure published by Sasaki et al. [28]. Binding assays were performed in triplicates in 0.02 M Tris-HCl, 0.11 M NaCl containing 0.05% P-20 surfactant (Biacore AB) at a flow rate of 20 μl/min. The association phase was monitored for 3 min, and the dissociation curve was recorded for 10 min. The bulk effects were subtracted using the reference control surfaces. The chips were regenerated by treatment with 1 M NaCl for 30 s. For the calculation of kinetics constants, the sensorgrams at concentrations of 0, 10, 50, 200, and 400 nM were fitted globally to the 1:1 Langmuir model with BIAevaluation software version 3.1.

ELISA assays were based on a procedure used by Rehn et al. [25]. Laminin, type IV collagen, nidogen-1, fibulin-1, and 10 µg/ml heparin-BSA were coated onto the surface of microtiter wells at 4 °C overnight. All the other steps were performed at room temperature. The wells were blocked with 5% nonfat milk in 0.05 M Tris-HCl, pH 7.4, 0.11 M NaCl, 2 mM CaCl_2_, 1 mM MgCl_2_ (TBS-Ca/Mg) for 1 h and then washed with 0.05% Tween-20 in TBS-Ca/Mg, and incubated for 3 h with endostatin as a soluble ligand diluted in 5% nonfat milk TBS-Ca/Mg. After thoroughly washing, the samples were incubated for 1 h with the antibody HES.6 against the human endostatin domain [29] diluted in TBS-Ca/Mg-5% milk, followed by washing with 0.05% Tween-20 in TBS-Ca/Mg, and incubation with the secondary antibody conjugated with horseradish peroxidase. Endostatin bound to the immobilized proteins was detected by adding 5-aminosalicylic acid (Sigma) in the presence of 0.01% H_2_O_2_. Detection was performed at 490 nm.

## Results

### Identification of new mutations in *COL18A1* leading to KS

Mutation screening of the *COL18A1* gene was performed in five KS patients, who were from four unrelated families (KS14–17). All the patients presented with congenital high myopia and occipital encephalocele, typical KS phenotypic characteristics. Three of the families are Brazilian (KS14, KS16, and KS17) and one is North American (KS15).

We identified four mutations not previously described: one insertion, two out-of-frame deletions in exons coding the C-terminal region of the protein, and a splice site mutation. All of these possibly lead to premature stop codons (Table 1). The mutation that occurs at the acceptor splice site of intron 7, c.929–2A>G, involves a highly conserved base and is predicted to disrupt splicing according to computational analysis (NetGene2 version 2.4 [30]; NNSPLICE version 0.9 [31]). We have detected a mutation in only one of the alleles in families KS14 and KS15, and these patients may be compound heterozygotes. Even though the KS16 patient was initially identified as a homozygous carrier of the mutation c.3514_3515delCT, we found only her mother harbors this mutation. Paternity was confirmed with the analysis of five microsatellite markers (D22S944, S1623, S1638, S1648, S1709). This patient also appeared homozygous for another mutation in this exon (c.3570G>A), which is present in the mother in heterozygosity but it is not present in the father, who carries only the G allele of this SNP. Therefore, we concluded that the father carries a deletion of at least exon 41 and the patient is actually hemizygous for the c.3514_3515delCT mutation and is a compound heterozygote for mutations in the *COL18A1* gene.

**Table 1:** Mutations identified in KS patients.

**Patient**	**Mutation**	**Region**	**Affected Isoforms**	**Description of mutation**
KS1	c.12–2A>T (homozygous)	Intron 1	Short isoform	[3]
KS3	c.2969–2978delCAGGGCCCCC (maternal)	Exon 36	All isoforms	[4]
	c.3514–3515delCT (paternal)	Exon 41		
KS4	c.1238–1239insA (maternal)	Exon 10	All isoforms	[4]
	c.3514–3513delCT (paternal)	Exon 41		
KS5	c.3514–3515delCT (maternal)	Exon 41	All isoforms	[4]
	c.2105delT (paternal)	Exon 23		
KS8	c.12–2A>T (homozygous)	Intron 1	Short isoform	[4]
KS9	c.3277C>T (homozygous)	Exon 40	All isoforms	[4]
KS10	c.2416C>T (homozygous)	Exon 18	All isoforms	[5], [17]
KS11	c.3769G>A (p.D1437N *) (maternal)	Exon 42	All isoforms	[15]
	c.2823_2824insC (paternal)	Exon 35		
KS12	c.3601G>A (p.A1381T *) (homozygous)	Exon 41	All isoforms	[18]
KS13	c.3544+3A>C (homozygous)	Intron 36	All isoforms	[16]
KS14	c.2673_2674insC (heterozygous)	Exon 33	All isoforms	Novel mutation
KS15	c.2824_2831delGGCCCCCC (heterozygous)	Exon 35	All isoforms	Novel mutation
KS16	c.3514–3515delCT (maternal)	Exon 41	All isoforms	Novel mutation
	exon 41 deleted (paternal)			Novel mutation
KS17	c.2673_2674insC (maternal)	Exon 33	All isoforms	Novel mutation
	c.929–2A>G (paternal)	Intron 7		Novel mutation

List of published COL18A1 mutations identified in Knobloch syndrome patients and the exon numbers of the mutation sites. Asterisk represents position based on the NC11–493 isoform (AF018081.1).

### p.A1381T mutation leads to a weaker binding affinity to some of the ECM proteins

**Figure 1:**
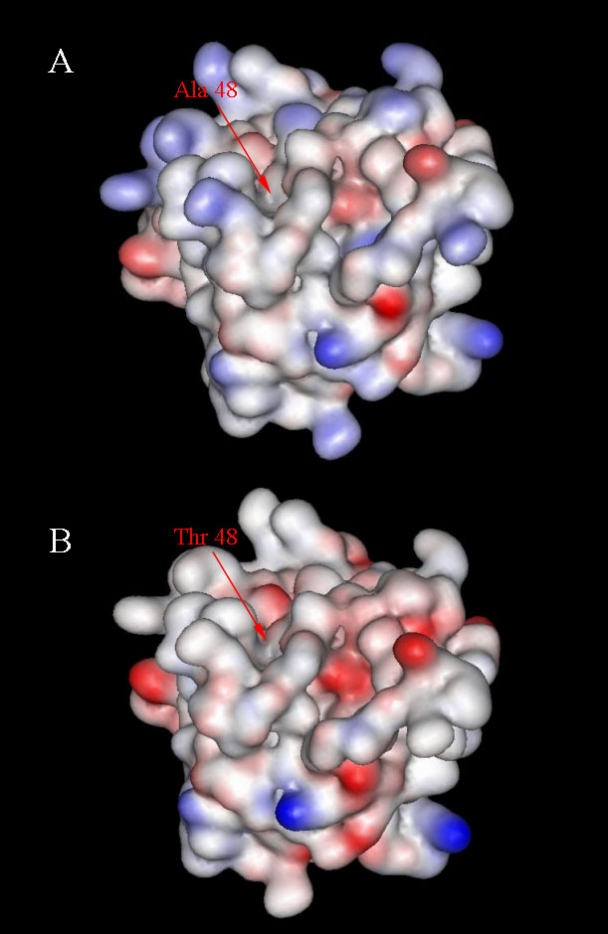
Molecular modeling of wild type and p.A1381T endostatins. Analysis of the electrostatic surface of wild type (**A**) and p.A1381T (**B**) endostatins. Areas shown in red represent a negative potential while the blue areas are positive. The position 48 of the endostatin domain (Protein Data Bank entry 1BNL) corresponds to the position 1381 of the NC11–493 isoform (GenBank AF018081.1).

**Figure 2:**
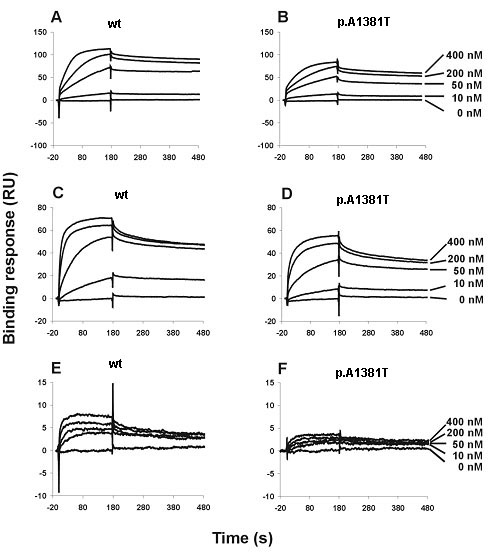
SPR sensorgrams of endostatin interactions with ECM components. Endostatin variants (wild type, mutant p.A1381T) were diluted to a concentration series in 0.02 M Tris-HCl, 0.11 M NaCl containing 0.05% P-20, and then injected into the sensor chips immobilized with laminin-1-nidogen-1 complex (**A**=wt, **B**=p.A1381T), fibulin (**C**=wt, **D**=p.A1381T), and perlecan (**E**=wt, **F**=p.A1381T) at 25 °C with a flow rate of 20 μl/min. Sensorgrams show binding of various concentrations of endostatin to the coated sensor surfaces. The association curves were monitored for 3 min, and the dissociation phases were recorded for 10 min but presented for 5 min. All the kinetics studies were performed three times independently at concentrations of 0–400 nM, and the data were analyzed with BIAevaluation software version 3.1 using the 1:1 Langmuir binding model.

To better understand if the rare p.A1381T endostatin mutation has the potential to disrupt its function, we performed ELISA and SPR assays to evaluate the fragment's ability to bind to other ECM proteins. Our molecular modeling analysis of the p.A1381T endostatin suggested that it was possible to accommodate the threonine side chain in this position (Figure 1). ELISA assays showed the binding properties of the p.A1381T endostatin to be similar to the wild type endostatin, without any significant differences between them. SPR experiments showed that the p.A1381T mutation leads to a somewhat weaker binding affinity to some of the ECM proteins that were tested (Figure 2; Table 2). The differences between wild type and mutant endostatin binding to laminin-1-nidogen-1 complex and to fibulin-1 were small but significant (p=0.0017 and p=0.0011, respectively). Perlecan binding was weak for both p.A1381T and wild type endostatins and the difference was not significant.

**Table 2:** Binding affinities (K_D_) of wild type and mutant endostatins to ECM components

**Sample**	**Laminin-1-nidogen-1 complex**	**Fibulin**	**Perlecan**
Wild type	3.48±0.24 nM	3.10±0.39 nM	5.53±1.53 nM
p.A1381T	6.17±0.27 nM	6.95±0.25 nM	5.19±0.92 nM

The kinetics constants were obtained by fitting the sensorgrams to the 1:1 Langmuir model.

### Skin biopsies from KS patients do not show *COL18A1* expression

**Figure 3:**
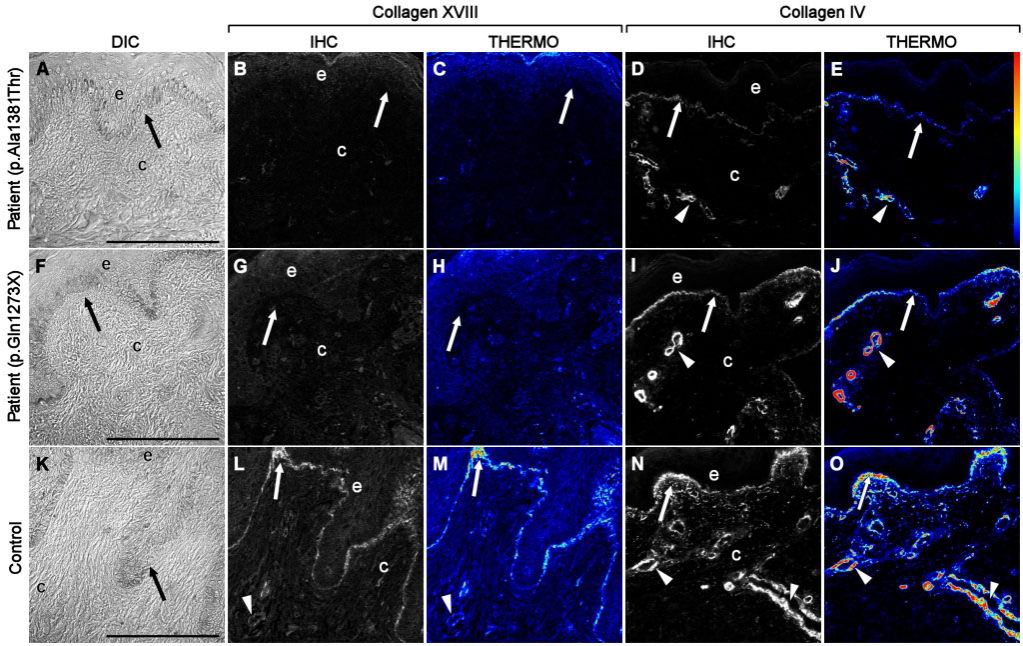
Immunofluorescent localization of type XVIII collagen in KS and control skin samples. Differential interference contrast (DIC) images (**A, F, K**) show that the epithelial and connective tissues obtained from skin biopsies were intact and well preserved after being prepared using cryofixation and cryosubstitution techniques. Immunolocalization analyses of KS patients carrying the p.A1381T change (**B, C**) and the nonsense mutation c.3277C>T (NC11–493 isoform position p.G1273X, GenBank AF018081.1; **G, H**) were negative for expression of collagen XVIII as compared to controls (**L, M**). Pseudocolor, thermo images showed that collagen XVIII expression was present in control samples (**M**) but undetectable in KS samples (**C, H**). Although the distribution pattern of type IV collagen was similar in both control (**N, O**), and KS (**D, E, I, J**) groups, the immunostaining intensity was noticeably lower in KS samples. The thermo color bar to the right of panel **E** indicates immunofluorescence staining intensity: red, higher levels; blue/black, lower levels. Arrows point to basement membrane, and arrowheads mark blood vessels. Scale bars equal 100 μm.

Null mutations that affect all type XVIII collagen isoforms must lead to complete lack of the protein. To confirm the absence of type XVIII collagen and to evaluate the possibility of distinguishing KS patients from normal individuals by the analysis of type XVIII collagen in skin biopsies, we performed fluorescent immunohistochemical staining for type XVIII collagen in skin biopsies from two KS individuals carrying the previously described nonsense mutation c.3277C>T. We observed a complete lack of type XVIII collagen expression in the tissue sections, while control samples displayed high levels of staining in the epithelial and endothelial basement membranes (Figure 3). The lack of expression of type XVIII collagen was especially evident in pseudocolor, thermo images in which even minor amounts of immunofluorescently labeled material can be visualized. Although another basement membrane protein, type IV collagen, showed a similar distribution pattern in both control and KS groups, its immunostaining intensity in KS samples was noticeably lower (Figure 3).

To understand the cause of KS in individuals carrying the change p.A1381T we also performed immunohistochemistry from skin biopsies of two patients. Surprisingly, they also showed lack of type XVIII collagen staining (Figure 3).

## Discussion

KS is thought to be a rare condition: only 42 patients from 15 unrelated families have been described [2,4,5,16-18,20,32-36]. Among these families, only 12 different *COL18A1* mutations have been identified [3-5,15-17]. Although some clustering has been observed in exon 41, there is heterogeneity of mutation site distribution. Here we describe four unrelated families harboring five different mutations (Table 1); all of the patients are compound heterozygotes. KS16 represents an unusual case, where the chromosome inherited from the father has an undetermined deletion in *COL18A1* that includes exon 41. This mechanism may elucidate cases already reported where *COL18A1* mutations were not detected [4]. Sequencing the 43 exons of this gene is laborious for small study centers to confirm the KS diagnosis, so the identification of biases in mutation distribution could help in the molecular diagnosis. Mutations identified so far indicate that exons 30 through 42 of *COL18A1* are more frequently mutated, as 5 (83.3%) out of 6 mutations here identified were located in these exons, as well as 9 (64.3%) out of 14 previously identified mutations. Interestingly, 5 (26.3%) out of 19 mutations previously identified are located in exon 41, pointing to this exon as the most frequently affected. For this reason, we suggest that mutation screening in KS patients should start with these exons. It is possible that KS is less rare, and the spectrum of clinical variability is wider than predicted.

To find an additional way to detect type XVIII collagen disruption in KS patients and gain a deeper understanding of the KS etiology, we performed protein measurements in situ and characterization of a mutation with an unknown effect in vitro. We evaluated for the first time the immunohistochemical presence of type XVIII collagen in KS patients. We have analyzed the distribution pattern of type XVIII collagen in skin biopsies of four KS patients: two siblings with the known pathogenic nonsense mutation c.3277C>T and two other siblings with the missense endostatin mutation c.3601G>A (p.A1381T). As expected for carriers of null mutations, we were unable to detect type XVIII collagen in any of the tested KS patients, including those with typical KS clinical features that harbor the mutation p.A1381T, of uncertain effect, confirming that the primary molecular defect is in collagen XVIII. Interestingly, the KS samples also show a lower expression level of type IV collagen when compared with the control samples. Since endostatin/collagen XVIII binds to several ECM components [12-14], it is possible that a lack of this protein may lead to changes in the overall organization and stability of the ECM, especially in the basement membrane where it is normally expressed.

We also tested the p.A1381T endostatin for binding affinity to a selection of ECM proteins. Even though the molecular modeling of the protein did not predict a significant modification, p.A1381T has a weaker affinity to some of the tested ECM components when compared with wild-type endostatin. Indeed, Stahl et al. (2005) [19] used a recombinant human p.A1381T endostatin in a mammalian expression system and suggested that this change leads to a reduced folding efficiency. They based this suggestion on the residue position and the observed 40% reduction in p.A1381T endostatin production. Considering that KS is probably caused by a complete disruption of at least the isoform NC11–303, the current data does not allow us to conclude that the p.A1381T residue change is sufficient to lead to the KS phenotype or is pathogenic, but this hypothesis seems unlikely. Although in silico analysis indicates that the c.3601G>A change does not create new splice sites (data not shown), we cannot exclude the possibility that it affects splicing in vivo and leads to creation of a premature stop codon, since no type XVIII collagen was detected in the skin biopsies of these patients. It is also possible that the KS in this family is caused by a yet to be detected mutation or larger deletion in *COL18A1.*

We have previously shown that endostatin serum levels measured by an ELISA assay were lower in KS patients as compared with controls [4]. Although we were able to discriminate patients and controls through this method, we raised the hypothesis that the antibody used in the assay is cross-reactive. This is evidenced by the positive endostatin levels in KS patients that are carriers of null mutations, which should lead to a complete or nearly complete lack of type XVIII collagen. The immunohistochemistry results confirm our prediction that null mutations in the *COL18A1* gene lead to a severe depletion of the protein. Therefore, immunohistochemical assessment of type XVIII collagen for diagnosing KS may be more precise than endostatin measurements through ELISA assays. The mutation screening of *COL18A1* gene is still expensive and laborious, and the effect of some detected mutations may be uncertain, as is the case for the endostatin p.A1381T change. Even when a possible pathogenic mutation is found, the immunohistochemical analysis of skin biopsies can be an important tool to confirm the diagnosis. Finally, we show for the first time that it is possible to diagnose KS through the direct detection of the protein. In turn, this could allow the screening of a larger number of patients with a possible diagnosis of KS and could help in understanding the phenotypic spectrum of this syndrome.
